# Integration of transcriptomics and metabolomics reveals the molecular mechanisms underlying the effect of nafamostat mesylate on rhabdomyolysis-induced acute kidney injury

**DOI:** 10.3389/fphar.2022.931670

**Published:** 2022-11-29

**Authors:** Wenli Guo, Yu Wang, Yuxuan Wu, Jiang Liu, Ying Li, Jing Wang, Santao Ou, Weihua Wu

**Affiliations:** ^1^ Metabolic Vascular Disease Key Laboratory, Sichuan Clinical Research Center for Nephropathy, Department of Nephrology, Affiliated Hospital of Southwest Medical University, Luzhou, Sichuan, China; ^2^ Department of Nephrology and Rheumatology, Sichuan Provincial People’s Hospital Qionglai Hospital, Medical Center Hospital Of Qionglai City. Chengdu, Sichuan, China

**Keywords:** rhabdomyolysis, acute kidney injury, nafamostat mesylate, transcriptomics, metabolomics, glutathione metabolism, inflammation, ferroptosis

## Abstract

**Objective:** To investigate the role and mechanisms of action of nafamostat mesylate (NM) in rhabdomyolysis-induced acute kidney injury (RIAKI).

**Methods:** RIAKI rats were assigned into control group (CN), RIAKI group (RM), and NM intervention group (NM). Inflammatory cytokines and proenkephalin a 119–159 (PENKID) were assessed. Cell apoptosis and glutathione peroxidase-4 (GPX4) were detected using TUNEL assay and immunohistochemical staining. Mitochondrial membrane potential (MMP) was detected by JC-1 dye. The expression of genes and metabolites after NM intervention was profiled using transcriptomic and metabolomic analysis. The differentially expressed genes (DEGs) were validated using qPCR. The KEGG and conjoint analysis of transcriptome and metabolome were used to analyze the enriched pathways and differential metabolites. The transcription factors were identified based on the animal TFDB 3.0 database.

**Results:** Serum creatinine, blood urea nitrogen, and PENKID were remarkably higher in the RM group and lower in the NM group compared to the CN group. Pro-inflammatory cytokines increased in the RM group and notably decreased following NM treatment compared to the CN group. Tubular pathological damages were markedly attenuated and renal cell apoptosis was reduced significantly in the NM group compared to the RM group. The expression of GPX4 was lower in the RM group compared to the CN group, and it increased significantly after NM treatment. A total of 294 DEGs were identified in the RM group compared with the NM group, of which 192 signaling pathways were enriched, and glutathione metabolism, IL-17 signaling, and ferroptosis-related pathways were the top-ranking pathways. The transcriptional levels of *Anpep, Gclc*, *Ggt1*, *Mgst2*, *Cxcl13*, *Rgn*, and *Akr1c1* were significantly different between the NM and RM group. *Gclc* was the key gene contributing to NM-mediated renal protection in RIAKI. Five hundred and five DEGs were annotated. Compared with the RM group, most of the upregulated DEGs in the NM group belonged to Glutathione metabolism, whereas most of the downregulated DEGs were related to the transcription factor Cytokine-cytokine receptor interaction.

**Conclusion:** NM protects the kidneys against RIAKI, which is mainly associated with NM mediated regulation of glutathione metabolism, inflammatory response, ferroptosis-related pathways, and the related key DEGs. Targeting these DEGs might emerge as a potential molecular therapy for RIAKI.

## Introduction

Rhabdomyolysis (RM) is a clinical syndrome of a muscular disorder caused by trauma, stroke, major artery occlusion, status epilepticus, genetic defects, infections etc. Rhabdomyolysis is characterized by the release of skeletal muscle-cell contents, such as myoglobin, sarcoplasmic proteins, and electrolytes into circulation (Shapiro et al., 2012; McMahon et al., 2013). Elevated myoglobin indicates the severity of RM (Bagley et al., 2007), which can result in life-threatening complications, including acute kidney injury (AKI), hyperkalemia, hypocalcemia or hypercalcemia, metabolic acidosis, hyperuricemia, hyponatremia, hypovolemic shock, and cardiac dysrhythmias (Bosch et al., 2009; Cervellin et al., 2010). It is worth noting that RM induced AKI (RIAKI) occurs in 19%–58% of patients with rhabdomyolysis and is associated with high mortality (Simpson et al., 2016).

The pathophysiology of RIAKI is thought to be associated with the elevation of circulating myoglobin. Under normal physiological conditions, myoglobin binds to plasma globulins and only a very small portion of myoglobin is released into circulation and reaches the glomeruli. However, in severe muscle damage, the circulating myoglobin overwhelms the globulins’ binding capacity, which enables the free myoglobin to reach the renal glomeruli and tubules (Krouzecky et al., 2003; Petejova et al., 2014). The presence of myoglobin in the urine (known as myoglobinuria), leads to tubular cast formation and intratubular obstruction, accumulation of iron, and proximal tubular cell injury. It has been shown that myoglobin can bind to the Tamm-Horsfall protein in the tubular cast, particularly in an acidic environment. The cast-bound form of myoglobin results in oxidative stress, which leads to vasoconstriction and a significant reduction of renal cortical blood flow (Moore et al., 1998; Giannoglou et al., 2007). Another factor of pathogenesis of RIAKI is volume depletion caused by sequestration of fluids distributed into injured muscles. Cellular constituents released from damaged muscles results in metabolic acidosis and the resulting aciduria further aggravates tubular injury due to low urinary pH and presence of globin and ferrihemate derived from myoglobin can cause a direct nephrotoxic effect (Chatzizisis et al., 2008).

Currently, there is no specific treatment for RIAKI. Dialysis remains the core treatment for patients with AKI secondary to rhabdomyolysis. It is hypothesized that dialysis can remove the circulating myoglobin released from injured muscles and metabolic toxic substances in the circulation. Continuous renal replacement therapy (CRRT) has emerged as one of the most important modalities for the prevention and treatment of RIAKI, especially for hemodynamically unstable patients (Vanholder et al., 2011). Due to the rapid repletion of plasma myoglobin following a single hemodialysis therapy, intermittent hemodialysis is usually ineffective in maintaining a low level of plasma myoglobin, while CRRT has greater clearance of myoglobin, and is more effective than conventional filters (Vanholder et al., 2011; Zimmerman and Shen, 2013). To ensure the safety and efficacy of CRRT, prolonged anticoagulation therapy is required to prevent clotting in the extracorporeal circuits. Heparin has been used frequently as an anticoagulant in the implementation of CRRT, but may increase the risk of hemorrhage and thrombocytopenia (Webb et al., 1995; van de Wetering et al., 1996). Nafamostat mesylate (NM) is a synthetic serine protease inhibitor, which exerts an inhibitory effect on various enzyme systems, such as the fibrinolytic, coagulation, and complement system (Maruyama et al., 1996). In 1990, NM was introduced into CRRT as an alternative anticoagulant in patients with a tendency of bleeding. Due to limited published data and insufficient evidence of safety and efficacy of NM in CRRT, the acceptance of NM as a standard alternative anticoagulant has been hampered and the use of NM was initially limited to Japan. However, in 2005, NM was licensed in Korea when citrate anticoagulation is unavailable and the application of NM in CRRT has been increasing ever since (Ohtake et al., 1991; Nakae and Tajimi, 2003). It has been reported that NM attenuates ischemia reperfusion induced renal injury in mice, which is associated with the inhibition of apoptosis and reduction of nitric oxide overproduction mediated by NM (Na et al., 2016). However, the role of NM in the context of RIAKI is still unknown. Therefore, our study aimed to investigate the effect of NM acting rather than a simple anticoagulant on the RIAKI, and decipher the molecular mechanisms underlying the action of NM in RIAKI using integrated transcriptomic and metabolomic analysis.

## Materials and methods

### Animals and drugs

Male Sprague Dawley (SD) rats (body weight: 226 g ± 13) were received from the animal experiment center of Southwest Medical University. The animals were housed in a facility with controlled humidity and temperature and 12 h dark/light cycle. Food and water were given *ad libitum* throughout the study. Glycerin was purchased from Soleibo Technology Co., Ltd. (Beijing, China). Nafamostat Mesylate was purchased from Durui Pharmaceutical Co., Ltd. (Jiangsu, China).

### Experimental protocol

Thirty-six male SD rats were randomly divided into three groups: Control group (CN, *n* = 12), RIAKI group (RM, *n* = 12), and NM intervention group (NM, *n* = 12). The NM group was further divided into two subgroups: 24 h and 48 h observation groups (*n* = 6 in each group). The CN group received an intramuscular injection of normal saline (10 ml/kg); the RM group received an intramuscular injection of 50% (v/v) glycerol normal saline (10 ml/kg); and NM group received intraperitoneal injection of 1 mg/kg NM 30 min prior to the modeling. The CN group and the RM group also received an intraperitoneally injection of the same amount of normal saline.

### Kidney function evaluation

Blood samples were collected without anticoagulants and centrifuged at 3,000 rpm for 10 min to harvest the serum. The serum blood urea nitrogen (BUN) and serum creatinine (SCr) were measured using an autoanalyzer (Siemens, Germany).

### Enzyme-linked immunosorbent assay assessment

The Rat interleukin (IL)-6 ELISA kit, rat tumor necrosis factor *α* (TNF-α) kit (Enzyme Immunoassay Industry Co., Ltd., China), and rat proenkephalin (PENKID) kit (Enzyme Immunoassay Industry Co., Ltd., China) were used to measure rat serum IL-6, TNF-α, and PENKID according to the manufacturer’s protocol. Absorbance levels of the inflammatory cytokines’ concentrations were detected at 450 nm using a microplate reader.

### Histopathological evaluation

Kidney tissue was fixed in 10% (v/v) formalin, embedded in paraffin, and sectioned at 4 μm thickness. Following sectioning, H&E staining was performed. The extent of tubular vacuolation, dilatation, and necrosis were evaluated. Renal tubular damage was assessed using the following scoring system: 0 represents no tubular injury; 1 represents less than 25% tubular injury; 2 represents 25%–50% tubular injury; 3 represents 51%–75% tubular injury, and 4 represents more than 75% tubular injury. Five fields were randomly selected from each slide for assessments of renal tubular injury.

### Terminal deoxynucleotide transferase dUTP nick end labeling staining

Apoptosis in kidney sections was detected by terminal deoxynucleotide transferase dUTP nick end labeling (TUNEL) assay using the TUNEL apoptosis detection kit (Roche, Switzerland) according to the manufacturer’s instructions. The number of TUNEL-positive cells and the total cells in 5 randomly selected fields of each slice were calculated as the apoptosis index (AI).

### Measurement of mitochondrial membrane potential

Mitochondrial membrane potential (MMP) was detected by JC-1 dye (Haimen BiYunTian Biotechnology Research Institute, Nantong, China) according to the manufacturer’s instructions. JC-1 aggregates in mitochondria in normal health cells, fluorescencing red, while in apoptotic cells, JC-1 accrues in the cytosol, as a green fluorescencing monomer. In brief, harvested cells from kidney tissue were incubated with 500 µl of JC-1 working solution in incubator for 20 min, washed, and resuspended with 500 µl of assay buffer, and analyzed by flowcytometry (cytoflex, Beckman).

### Immunohistochemical staining

Kidney sections were deparaffinized, hydrated, and antigen-retrieved. Endogenous peroxidase activity was quenched by incubating the specimens with a 3% (v/v) H_2_O_2_. The specimens were then blocked with a serum block solution (DAKO, Tokyo, Japan), followed by incubation with the primary antibody (Rabbit monoclonal anti-GPX4; Cat. No. ab231174; Abcam) overnight at 4°C in a humidified box. After incubation with the secondary antibody (Goat Anti-Rabbit IgG ab6721, Abcam) for 30 min at room temperature, sections were incubated with DAB reagents for coloration (Abcam). Slides were examined using a Nanozoomer S60 digital slice scanning system. The positive areas of IHC staining (brownish yellow) were analyzed. Average optical density was used to assess the expression/signal intensity of GPX4.

### Assessment of iron concentration and the GSH/GSSG ratio

The iron concentration of kidney tissue was measured by tissue iron assay kit (A039-2-1, Nanjing Jiancheng Bioengineering, China) according to the manufacturer’s protocol. GSH/GSSG ratio of kidney tissue was determined using total glutathione/Oxidized glutathione assay kit (A061-2-1, Nanjing Jiancheng Bioengineering, China) according to the manufacturer’s instruction.

### RNA isolation and transcriptomic analysis

Total RNA was isolated using TRIzol reagent (Invitrogen, CA, United States). The RNA concentration and quality were evaluated using a Nanodrop 2000 instrument (Thermo Scientific, United States) and an Agilent 2100 bioanalyzer (Agilent Technologies, Santa Clara, CA, United States). The RNA-seq libraries were prepared using Illumina Stranded mRNA LT Sample Prep Kit (Illumina, San Diego, CA, United States) according to the manufacturer’s instructions. The transcriptome sequencing and analysis were conducted by OE Biotech Co., Ltd (Shanghai, China).

After removing the low-quality sequence reads and the adapters with fastp^1^, the clean reads were mapped to the rat genome using HISAT2^2^. FPKM^3^ of each gene was calculated and the read counts of each gene were obtained by HTSeq-count^4^. PCA analysis was performed using R (v 3.2.0) to evaluate the biological duplication of samples.

Differential expression analysis was performed using the DESeq2^5^. A *p*-value <0.05 and foldchange >2 or foldchange <0.5 was set as the threshold for significantly differential expression gene (DEGs). Hierarchical cluster analysis of DEGs was performed using R (v 3.2.0) to demonstrate the expression pattern of genes in different groups and samples. The radar map of the top 30 genes was drawn to show the expression of upregulated or downregulated DEGs using R packet radar.

Based on the hypergeometric distribution, Gene Ontology^6^ and Kyoto Encyclopedia of Genes and Genomes (KEGG)^7^, pathway enrichment analysis of DEGs was performed to screen the significant enriched term using R (v 3.2.0), respectively. R (v 3.2.0) was used to draw the column diagram, the chord diagram and the bubble diagram of the significant enrichment term. Identification of transcription factors was conducted by matching DEGs with the rat transcription factors deposited in the Animal TFDB 3.0 database.

### RT-qPCR analysis

The same RNA samples used for transcriptomic analysis were also used for RT-qPCR analysis. The yield of RNA was determined using a NanoDrop 2000 spectrophotometer (Thermo Scientific, United States), and the integrity was evaluated using agarose gel electrophoresis stained with ethidium bromide.

Quantification was performed with a two-step reaction process: reverse transcription (RT) and PCR. Each RT reaction consisted of 0.5 μg RNA, 2 μl of 5 × TransScript All-in-one SuperMix for qPCR, and 0.5 μl of gDNA remover, in a total volume of 10 μl. Reactions were performed in a GeneAmp^®^ PCR System 9700 (Applied Biosystems, United States) for 15 min at 42°C, and for 5 s at 85°C. The 10 μl RT reaction mix was then diluted ten times in nuclease-free water and held at −20°C. RT-qPCR was performed using LightCycler^®^ 480 II Real-time PCR Instrument (Roche, Swiss) with 10 μl PCR reaction mixture that included 1 μl of cDNA, 5 μl of 2 × PerfectStartTM Green qPCR SuperMix, 0.2 μl of forward primer, 0.2 μl of reverse primer, and 3.6 μl of nuclease-free H_2_O. Reactions were incubated in a 384-well optical plate (Roche, Swiss) at 94°C for 30 s, followed by 45 cycles of 94°C for 5 s, and 60°C for 30 s. Each sample was run in triplicate for analysis. At the end of the PCR cycles, melting curve analysis was performed to validate the specific generation of the expected PCR product. Each sample was analyzed in three independent biological replicates, and the 2^−ΔΔCT^ method was used to determine the gene expression levels. The rat *ACTB* gene was used as an internal control. The primers were designed by OE Biotech Co., Ltd. (Shanghai, China), based on the mRNA sequences obtained from the NCBI database and synthesized by Tsingke Biotechnology Co., Ltd. (Beijing, China). Primers sequences are shown in Table 1.

**TABLE 1:** Primers sequences and characteristics.

Gene symbol	Primer	Product length (bp)	Gene ID
*Akr1c2*	Forward GAT​CTT​CCT​TGG​AAT​GTT​CAC​T	87	291,283
	Reverse CTGGTTTCAAGGCCATCG		
*Akr1c1*	Forward TTC​TGC​TGC​TGT​GTA​TCA​GAA​T	80	307,092
	Reverse CTC​TCT​TCA​CAG​TAC​CGT​C		
*Rgn*	Forward TGC​TGT​TTG​TAG​ACA​TCC​CT	82	25,106
	Reverse GCATCTACACCAACTCGC		
*Anpep*	Forward GGTTTGGCAACCTGGTGA	85	81,641
	Reverse GCA​CCC​AGA​AAT​TCC​ACA​TAG		
*Cxcl13*	Forward AAT​GTA​GGT​GTT​CCA​AGG​TGA	80	498,335
	Reverse ATT​CCC​AGG​GCG​TAT​AAC​T		
*Cxcl1*	Forward AGACAGTGGCAGGGATTC	93	81,503
	Reverse GTGTGGCTATGACTTCGG		
*Mgst2*	Forward GCAAGTCGGACGAGTAAG	86	295,037
	Reverse GTG​CGC​GAA​ATA​TTC​TCT​CAA		
*Gclc*	Forward CGA​TGT​CCG​AGT​TCA​ACA​C	85	25,283
	Reverse AGA​GCC​TGA​TGT​TCT​CCT​AA		
*ggt1*	Forward AAG​CGT​TGC​TCA​GAG​ATT​G	85	116,568
	Reverse CCATACACAGCAGGCTTG		
*Retn*	Forward TAC​TGC​CAG​CTG​CAA​TGA​A	96	246,250
	Reverse TAGTGACGGTTGTGCCTT		
*ACTB*	Forward GCG​AGT​ACA​ACC​TTC​TTG​C	72	81,822
	Reverse TAT​CGT​CAT​CCA​TGG​CGA​AC		

### Liquid chromatography–mass spectrometry metabolite measurements and metabolomic analysis

The metabolites in the kidney samples were extracted by following the established protocol (Leuthold et al., 2018). Liquid chromatography–mass spectrometry analyses were performed on a Dionex U3000 UHPLC system (Thermo, United States) coupled with a Q Exactive Plus mass spectrometer (Thermo, United States). The ACQUITY HSS T3 UPLC columns (Waters, United States) were used. The solvent B gradient was set as follows: 5% (0–2 min), 5%–30% (2–4 min), 30%–50% (4–8 min), 50%–80% (8–10 min), 80%–100% (10–14 min), 100%–100% (14–15 min), 100%–5% (15–15.1 min), 5%–5% (15.1–16 min). The mass spectrometer was operated in positive and negative modes. The raw data was processed by Progenesis QI v2.4 (Waters, United States) following steps of alignment of the chromatograms, baseline filtering, normalization, integration, peak recognition, and retention time correction. The *t*-test and the score of variable importance in projection (VIP) were used to screen the differential metabolites between groups. The differential metabolites were mapped to the KEGG database for pathway enrichment analysis.

### Combined metabolomic and transcriptomic analysis

Pearson correlation coefficients and *p* values were used to screen metabolites and related genes within the conjoint analysis of metabolomics and transcriptomics. To further understand the relationship between DEGs and differential metabolites, the DEGs and metabolites were mapped to their associated KEGG pathways.

### Statistical analysis

Statistical analysis was performed using IBM SPSS 25.0 statistical software (IBM Corp.). The data were expressed as mean ± standard deviation comparison between groups was performed by one-way ANOVA, and pairwise comparison between groups was performed by S-N-K test. A Kruskal-Wallis test was performed when data were heteroscedastic and a result of *p* < 0.05 was considered statistically significant. For the transcriptomic analysis, the DEGs were detected with a false discovery rate-adjusted *p*-value <0.05 and foldchange >2 or foldchange <0.5. For the metabolomic analysis, the threshold for the *t*-test was *p* < 0.05, and the threshold for VIP score was set at ≥ 1.

## Results

### The effect of nafamostat mesylate on renal function in rhabdomyolysis-induced acute kidney injury

**FIGURE 1:**
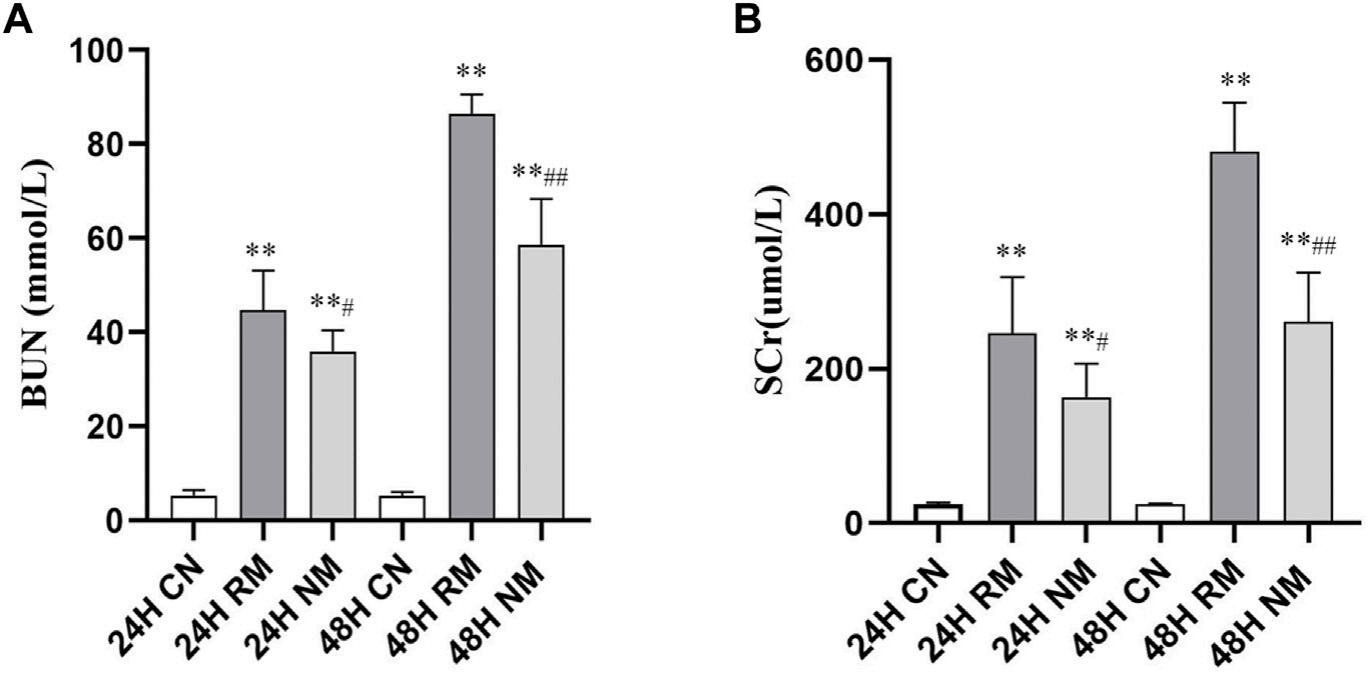
Effect of NM on serum BUN and SCr levels in RIAKI. **(A)** The effect of NM on glycerol induced changes in BUN and SCr. **(B)** The effect of NM on glycerol induced changes in SCr (*p < 0.05, and **p < 0.01 vs. CN; ^#^ *p* < 0.05, and ^##^ *p* < 0.01 vs. RM).

There was a significant increase in serum BUN and SCr in the RM group at both 24 h and 48 h compared to the CN group (*p* < 0.01). The levels of serum BUN and SCr decreased significantly in the NM group compared to the RM group at 24 h (*p* < 0.01), and there was a significant reduction in serum BUN and SCr following 48 h intervention of NM (*p* < 0.01) (Figure 1).

### Effect of nafamostat mesylate on inflammatory cytokines and proenkephalin in rhabdomyolysis-induced acute kidney injury

**FIGURE 2:**
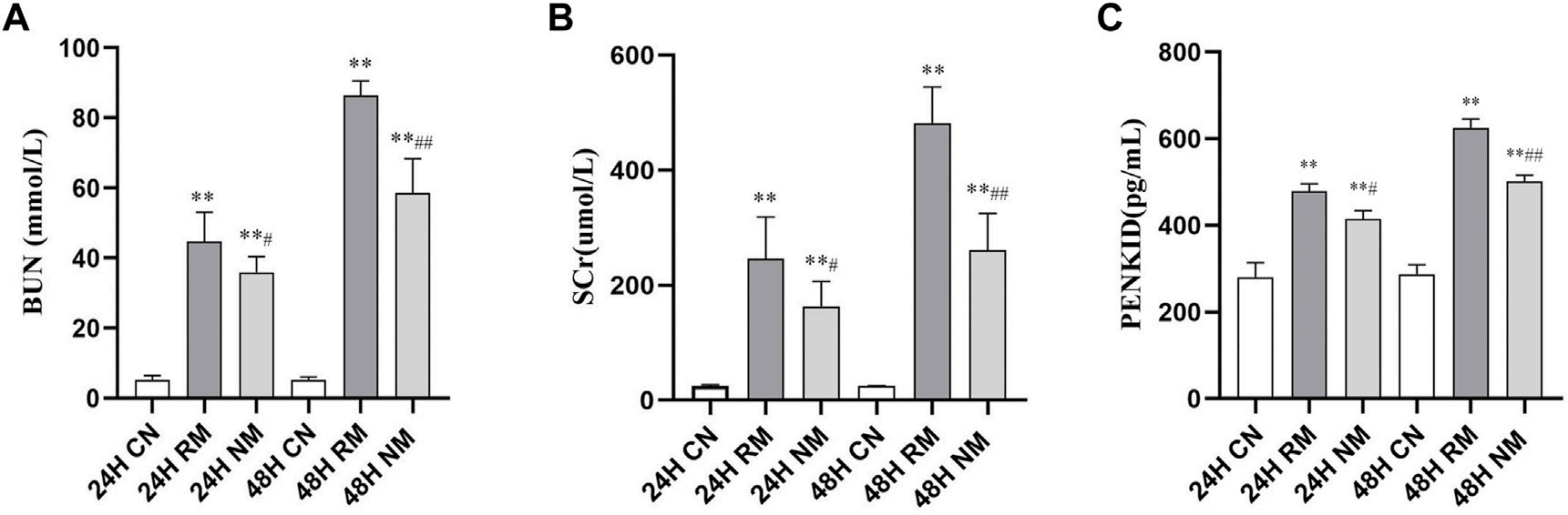
Effect of NM on serum levels of pro-inflammatory cytokines and PENKID. **(A)** The effect of NM on glycerol induced changes in IL-6. **(B)** The effect of NM on glycerol induced changes in TNF-α. **(C)** The effect of NM on PENKID (*p < 0.05, and **p < 0.01 vs. CN; ^#^ *p* < 0.05, and ^##^ *p* < 0.01 vs. RM).

There was a significant increase in the serum levels of IL-6, TNF-α and PENKID in the RM group compared to the CN group at both 24 h and 48 h after the glycerol challenge (*p* < 0.01). The level of serum IL-6 showed the trend of dropping at 48 h, while the level of serum TNF-α and PENKID continued to increase overtime. Nafamostat mesylate intervention significantly reduced the serum levels of IL-6, TNF-α and PENKID compared to the RM group (*p* < 0.01) (Figure 2).

### Effect of nafamostat mesylate on renal histopathology in rhabdomyolysis-induced acute kidney injury

**FIGURE 3:**
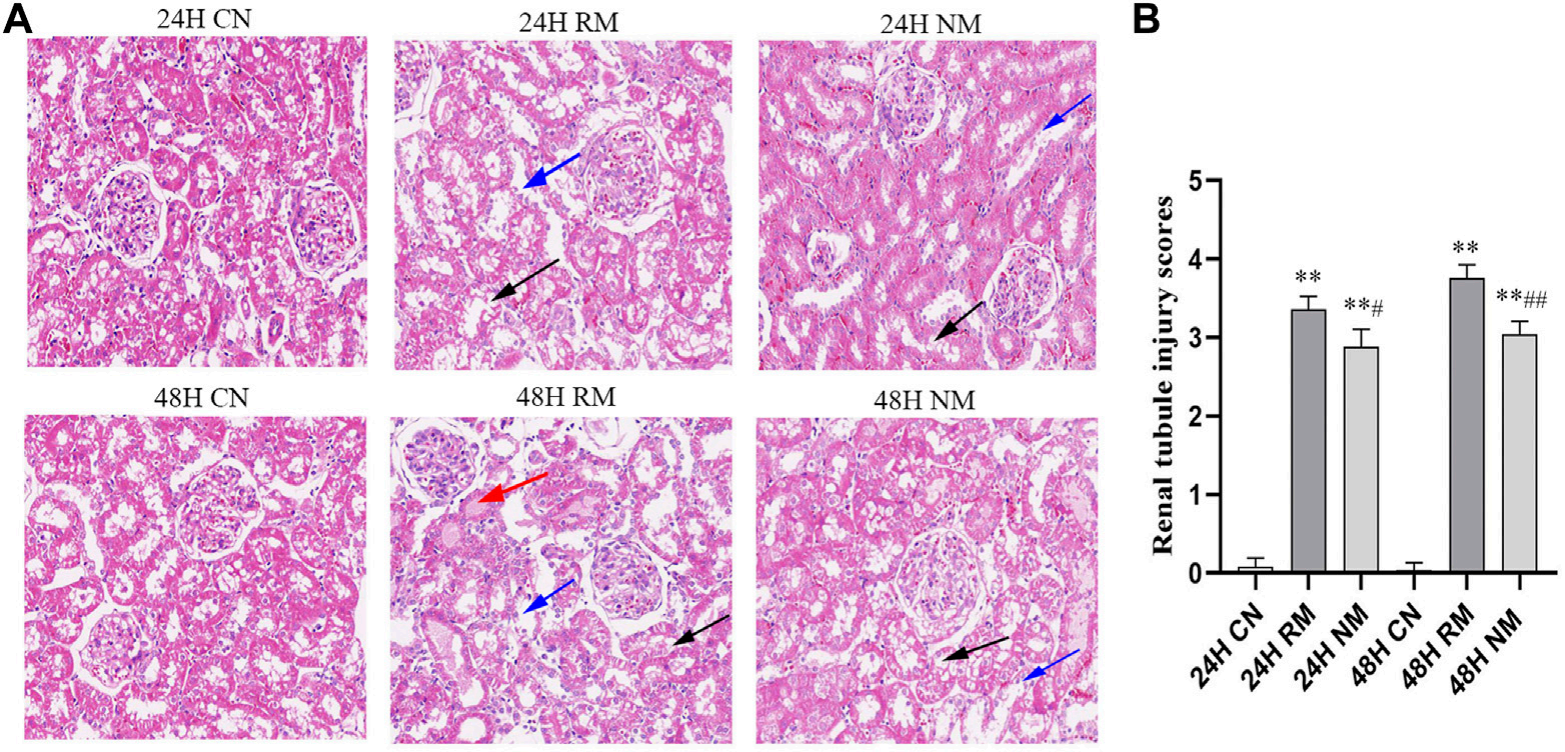
Effect of NM on renal histopathology in RIAKI. **(A)** Representative histological appearance of kidney tissue using H&E staining presented with magnification ×200. The black arrow indicates renal tubular necrosis and detachment; the blue arrow indicates renal tubular vacuole; the red arrow indicates the cast in the renal tubules. **(B)** Renal tubular injury scores (**p < 0.01 vs. CN; ^#^ *p* < 0.05, and ^##^ *p* < 0.01 vs. RM).

In H&E staining, a higher degree of vacuolization and tubular dilatation, and a large number of necrotic cell and myoglobin casts were noted in the RM group, whereas this phenomenon was mitigated with NM intervention. The renal tubular injury scores were significantly increased in the RM group compared with the CN group at both 24 h and 48 h time points (*p* < 0.01), while there was a significant decrease in renal tubular injury scores in the NM group compared with the RM group at 24 h (*p* < 0.05) and 48 h (*p* < 0.01) (Figure 3).

### Effect of nafamostat mesylate on renal tissue apoptosis in rhabdomyolysis-induced acute kidney injury

**FIGURE 4:**
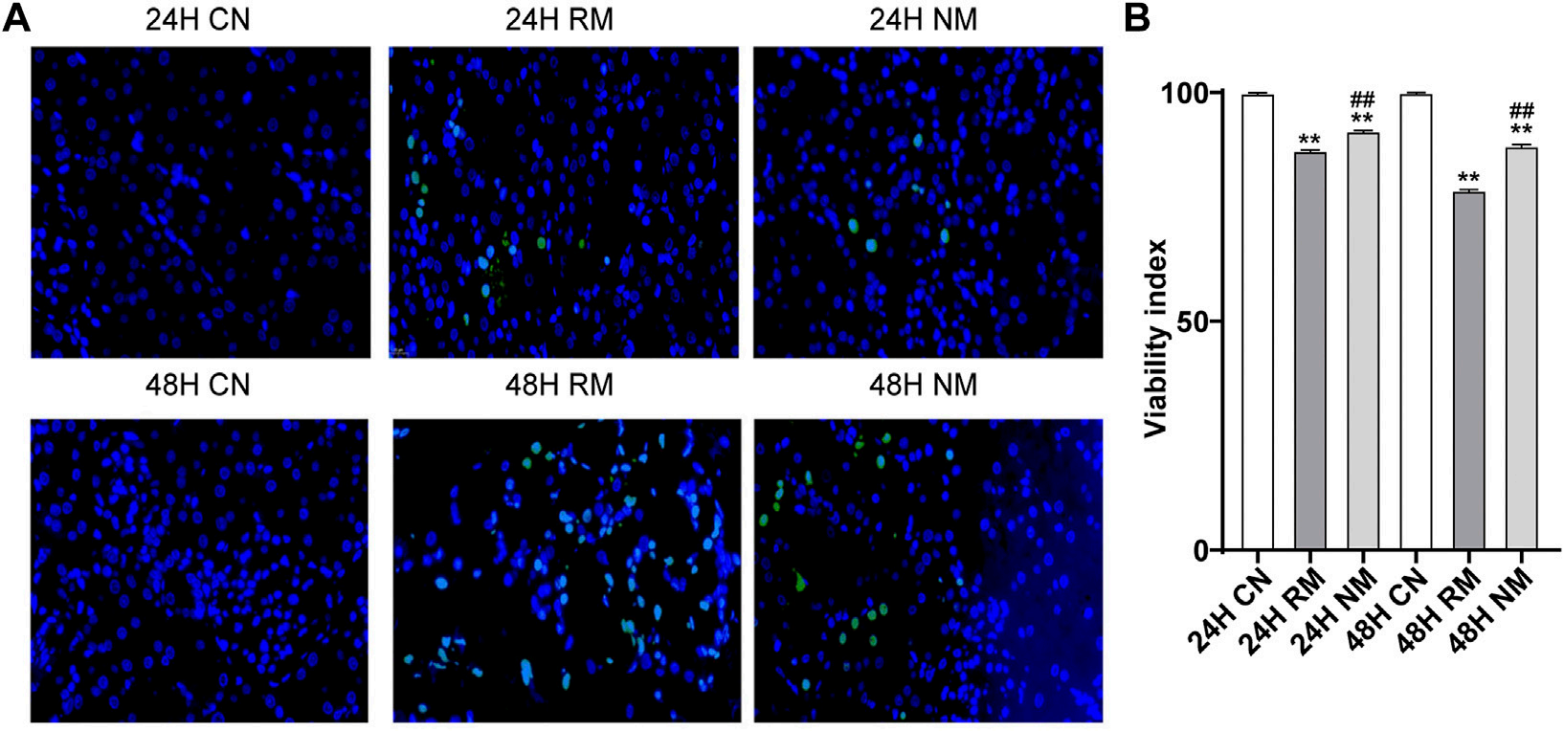
Effect of NM on renal tissue apoptosis in RIAKI. **(A)** Representative TUNEL staining images. **(B)** The AI (percentage of positive cells/neoplastic nuclei).

There were more TUNEL-positive cells in the RM group compared to the CN group at both 24 h and 48 h, while fewer TUNEL-positive cells were seen in the NM group compared with the RM group (Figure 4A). The AI staining showed that apoptosis was significantly increased in the RM group compared to the CN group at both 24 h and 48 h time points (*p* < 0.01), whereas it was mitigated significantly with NM intervention at both time points (*p* < 0.01) (Figure 4B).

### Effect of nafamostat mesylate on renal tissue ferroptosis in rhabdomyolysis-induced acute kidney injury

**FIGURE 5:**
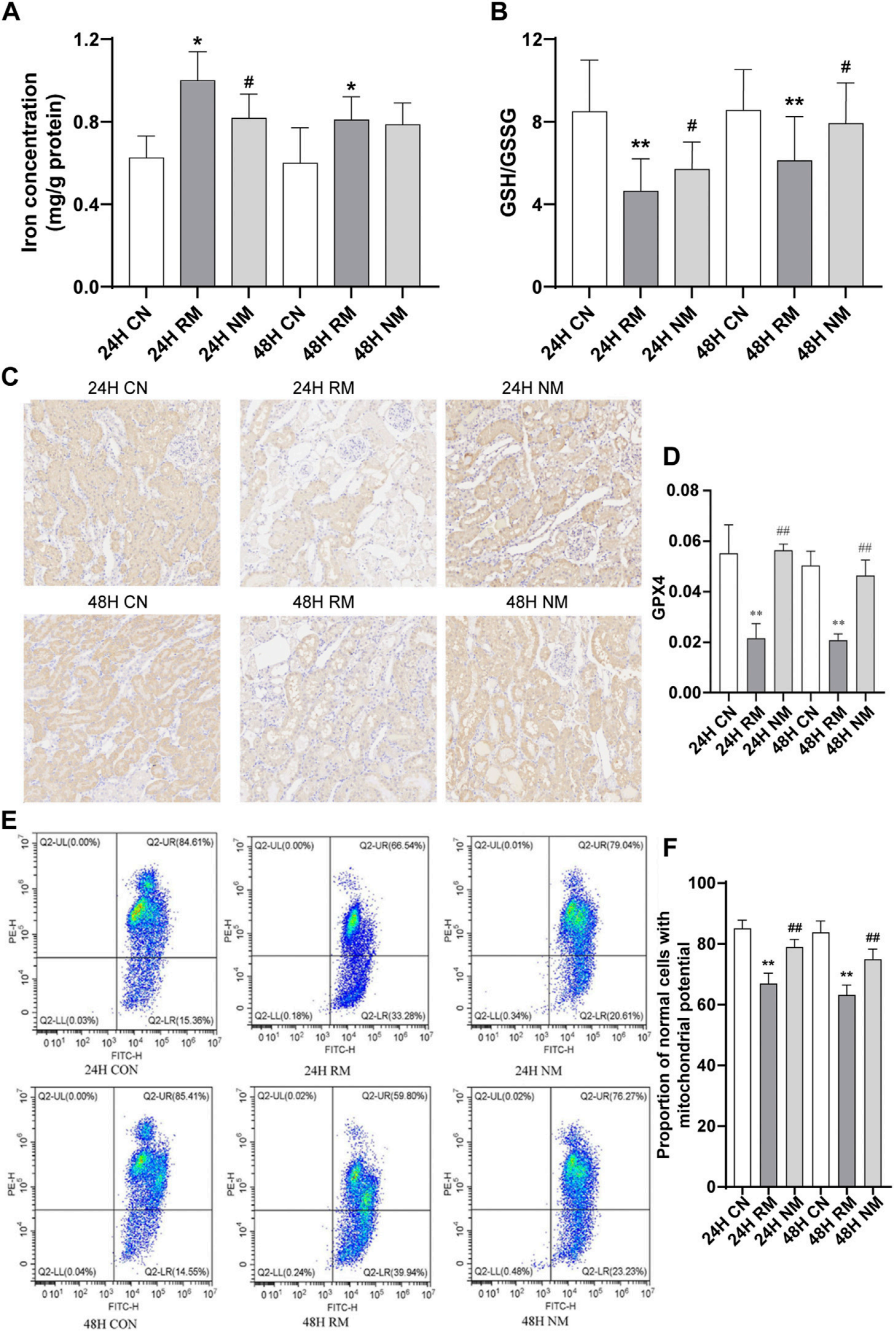
Effect of NM on renal tissue ferroptosis in RIAKI. **(A)** Iron level. **(B)** GSH/GSSG ratio. **(C)** Representative micrographs of immune staining of GPX4 in kidney tissues. Scale bar, 20 μm. **(D)** Semiquantitative analysis showing GPX4 expression in kidney tissues. **(E)** Representative images of flow cytometric assessment of changes in the MMP in response to RIAKI. **(F)** Quantification of ratio of JC-1 aggregates *versus* JC-1 monomers (**p < 0.01vs. CN; *p < 0.05 and ^##^ *p* < 0.01 vs. RM).

The iron accumulations increased significantly in the RM group at 24 h and 48 h compared to CN group at the corresponding time points (*p* < 0.05), and decreased after NM treatment at 24 h compared to RM group (*p* < 0.05). There is no significant difference in kidney tissue iron contents between NM and RM group at 48 h. The GSH/GSSG ratio showed a remarkable decrease in RM group compared to CN group at both time points (*p* < 0.01), while there was a significant increase in GSH/GSSG ratio after RM treatment at 24 h and 48 h time points compared to RM group (*p* < 0.05) (Figures 5A,B).

The expression of GPX4 was lower in the RM group compared to the CN group at both 24 h and 48 h (*p* < 0.01), while the abundance of GPX4 increased significantly after NM treatment (NM vs. RM, *p* < 0.01) (Figures 5C,D). The RM group showed an enhanced loss of MMP compared with the CN group, while NM treatment showed an improved loss of MMP compared with the RM group (*p* < 0.05) (Figures 5E,F). This suggests that the NM attenuates the loss in MMP in response to RIAKI.

### Differential gene expression profiles in rhabdomyolysis-induced acute kidney injury

**FIGURE 6:**
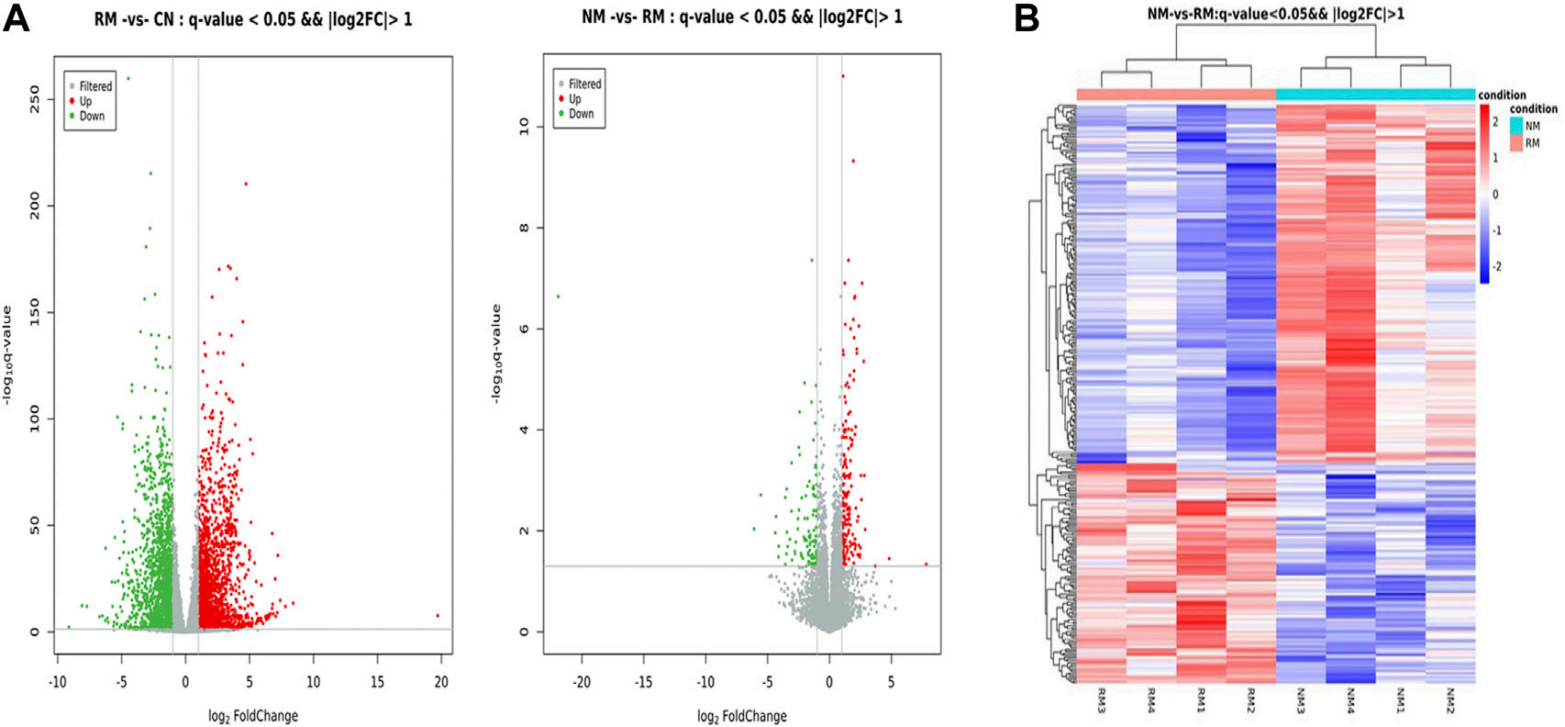
**(A)** Volcano plot of DEGs in RIAKI. The *x* and *y* axis represent log_2_ (fold change) and *p*-value, respectively. Red and green dots represent corresponding upregulated and downregulated genes, respectively. **(B)** Heatmap of hierarchical clustering of DEGs between NM and RM. Color intensity is proportional to the abundance of gene expression. Red represents upregulated genes and blue represents downregulated genes.

**FIGURE 7:**
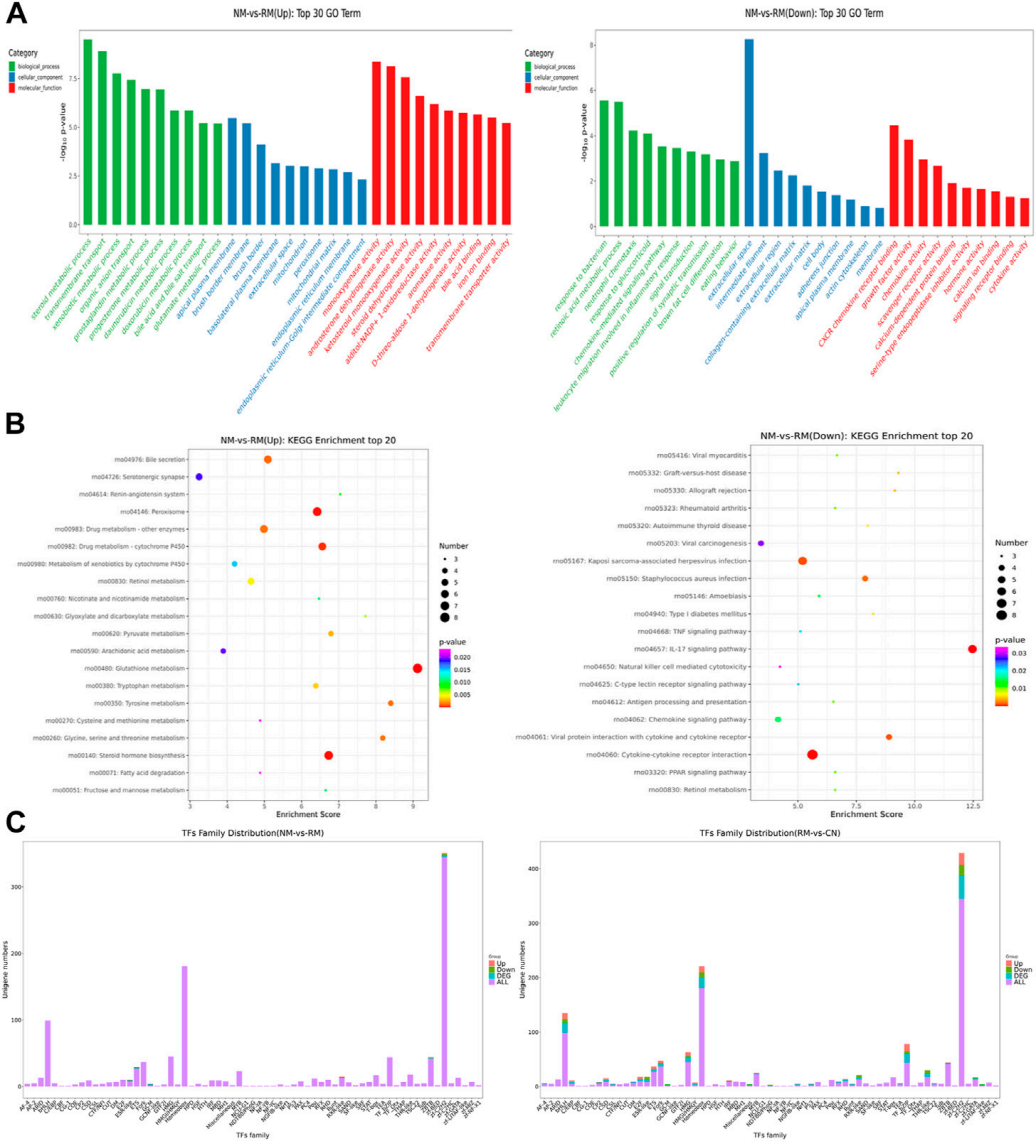
GO term and KEGG pathway enrichment analyses of DEGs. **(A)** GO annotations of DEGs were queried into functional groups based on enrichment scores. **(B)** The top 20 up or down regulated pathways are presented. The enrichment factor is the ratio of the number of DEGs to the number of total genes in a particular pathway. The dot size represents the number of DEGs, while colors correspond to the range of *p*-values. **(C)** Predicted transcription factors of DEGs. Red represents upregulated genes and blue represents downregulated genes.

The screening criteria for DEGs were a false discovery rate-adjusted *p* ≤ 0.05 and log2 fold change >1. The transcriptome analysis identified 3,891 DEGs in the RM group compared with the CN group, with 2,132 genes upregulated and 1,759 genes downregulated, and 294 DEGs in the NM group compared with the RM group, of which 182 genes were upregulated and 112 genes were downregulated (Figure 6). To gain further insight into related signaling pathways, KEGG pathway analysis was performed and revealed that 192 signaling pathways were enriched in the NM group compared with the RM group, of which 147 were up-regulated and 103 were down-regulated. The upregulated pathways were mainly implicated in glutathione metabolism, steroid biology synthesis, amino acid (tyrosine, glycine, serine, threonine, pyruvate, tryptophan, cysteine, and methionine) metabolism, arachidonic acid metabolism, metabolism of cytochrome P450-related substances, and peroxisome. The down-regulated pathways were classified in IL-17 signaling pathway, cytokine-cytokine receptor interaction, PPAR signaling pathway, chemokine signaling pathway, tumor necrosis factor signaling pathway, and bile secretion (Figures 7A,B). Five hundred and five DEGs (291 up and 214 down) were annotated with the transcription factor database. Compared with the RM group, the most of upregulated DEGs in NM group belonged to Glutathione metabolism, whereas the most of downregulated DEGs belonged to the transcription factor Cytokine-cytokine receptor interaction (Figure 7C). Notably, the glutathione and IL-17 signaling pathways were assigned the most DEGs.

### Verification of the key DEGs profiles in rhabdomyolysis-induced acute kidney injury

**FIGURE 8:**
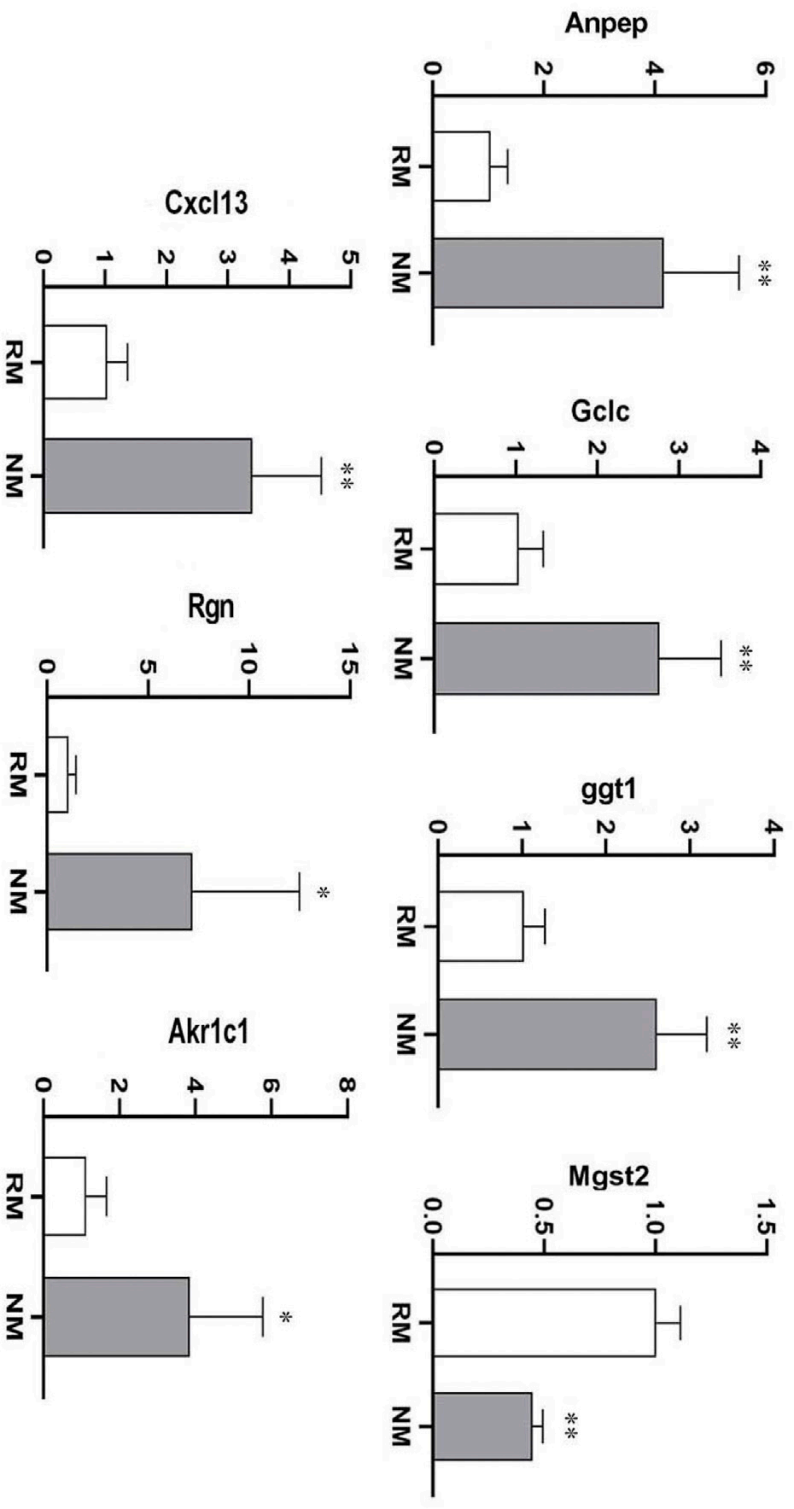
mRNA expression of key DEGs in RIAKI (*p < 0.05, **p < 0.01).

To further verify the credibility of the expression profiles of the key DEGs obtained from the RNA-seq data, 7 DEGs were selected based on the most enriched signaling pathways and a large fold change. The RT-PCR results showed that the expression trends of these DEGs were highly consistent with the transcriptome data. The nafamostat mesylate mediated glutathione metabolic pathway related genes *Anpep*, *Gclc*, *Ggt1* were upregulated, while *Mgst2* was significantly downregulated (*p* < 0.01). The inflammatory pathway related genes *Cxcl13* and *Rgn* were significantly upregulated (*p* < 0.01) and the ferroptosis pathway related gene *Akr1c1* was significantly upregulated (*p* < 0.01) (Table 2; Figure 8). Collectively, these results suggest that various biological pathways are involved in renal protection with NM in RIAKI.

**TABLE 2:** KEGG pathway enrichment analysis of DEGs (Top 20).

Pathway ID	Pathway	Gene ID	Score	*p*-value
rno00480	Glutathione metabolism	*Anpep*; *Gclc*; *Ggt1*; *Gsta2*; *Gsta5*; *Mgst2*; *Nat8*; *Nat8f1*; *Nat8f3*	7.21	<0.01
rno00140	Steroid hormone biosynthesis	*Akr1c1*; *Cyp21a1*; *Cyp2c11*; *Cyp2d1*; *Cyp2d2*; *Cyp2d4*; *Cyp2d5*; *Cyp3a9*	5.41	<0.01
rno00830	Retinol metabolism	*Adh6*; *Aldh1a3*; *Aox2*; *Cyp26b1*; *Cyp2c11*; *Cyp3a9*; *Cyp4a8*; *Rdh5*	5.22	<0.01
rno00982	Drug metabolism—cytochrome P450	*Adh6*; *Aox2*; *Fmo3*; *Fmo4*; *Gsta2*; *Gsta5*; *Mgst2*	5.38	<0.01
rno00350	Tyrosine metabolism	*Adh6*; *Aoc3*; *Aox2*; *Fahd1*; *Hgd*	7.38	<0.01
rno00260	Glycine, serine and threonine metabolism	*Aoc3*; *Cth*; *Dao*; *Gamt*; *Gnmt*	7.19	<0.01
rno04146	Peroxisome	*Amacr*; *Dao*; *Decr2*; *Eci3*; *Hao2*; *Mpv17l*; *Nudt12*	4.52	<0.01
rno00983	Drug metabolism—other enzymes	*Ces1f*; *Ces2h*; *Dpys*; *Gsta2*; *Gsta5*; *Mgst2*; *Upb1*	4.09	<0.01
rno04614	Renin-angiotensin system	*Anpep*; *Mcpt1l1*; *Mme*; *Ren*	6.60	<0.01
rno00760	Nicotinate and nicotinamide metabolism	*Aox2*; *Aspdh*; *Nnmt*; *Nudt12*	6.07	<0.01
rno00360	Phenylalanine metabolism	*Aoc3*; *Glyat*; *Glyatl1*	8.42	<0.01
rno04657	IL-17 signaling pathway	*Csf3*; *Cxcl1*; *Cxcl2*; *Cxcl6*; *S100a8*; *S100a9*	3.70	<0.01
rno04976	Bile secretion	*Aqp1*; *Slc22a7*; *Slc51a*; *Slco1a1*; *Slco1a6*; *Slco1b2*	3.58	<0.01
rno05150	*Staphylococcus aureus* infection	*C4a*; *Cfd*; *Krt15*; *Krt20*; *Krt23*; *Mbl1*	3.51	<0.01
rno05204	Chemical carcinogenesis	*Adh6*; *Cyp2c11*; *Cyp3a9*; *Gsta2*; *Gsta5*; *Mgst2*	3.47	<0.01
rno04060	Cytokine-cytokine receptor interaction	*Ackr4*; *Bmp2*; *Csf2rb*; *Csf3*; *Cxcl1 Cxcl13*; *Cxcl2*; *Cxcl6*; *Il1r2*; *Lep*; *Prlr*	2.29	<0.01
rno00620	Pyruvate metabolism	*Acss2*; *Adh6*; *Ldhd*; *Pc*	4.78	<0.01
rno04974	Protein digestion and absorption	*Col11a1*; *Col6a5*; *Mep1b*; *Mme*; *Slc36a2*; *Slc3a1*	3.27	<0.05
rno00071	Fatty acid degradation	*Acsbg1*; *Adh6*; *Cyp4a8*; *Eci3*	4.58	<0.05
rno00980	Metabolism of xenobiotics by cytochrome P450	*Adh6*; *Akr1c1*; *Gsta2*; *Gsta5*; *Mgst2*	3.69	<0.05

### Correlation of metabolic changes and gene expression in rhabdomyolysis-induced acute kidney injury

**FIGURE 9:**
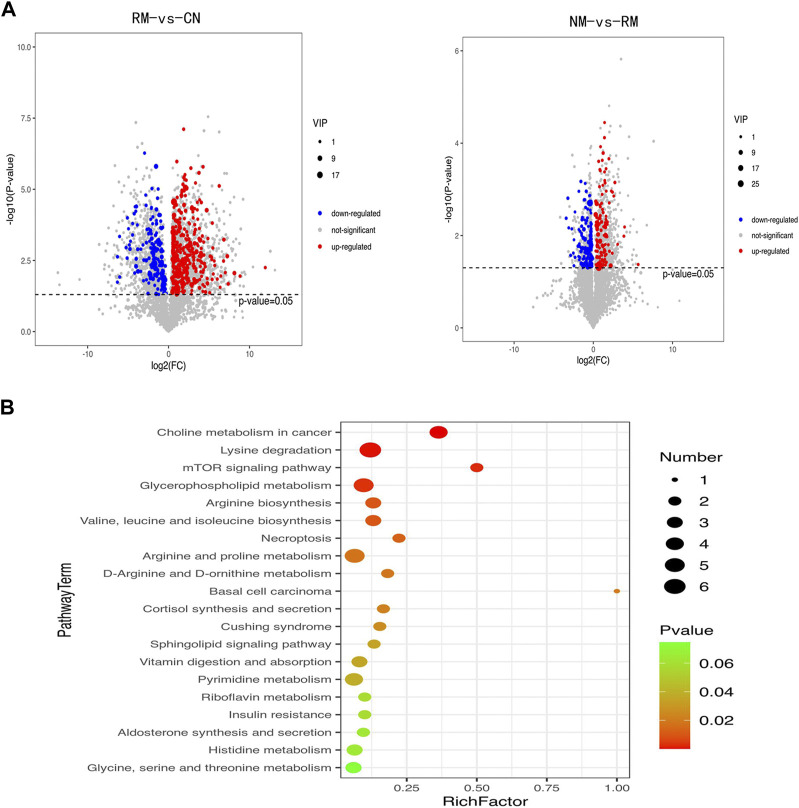
**(A)** Volcano plot of differential metabolites in RIAKI. *X* axis and *Y* axis represent log_2_ (fold change) and *p*-value, respectively. Red and green dots represent upregulated and downregulated metabolites, respectively. **(B)** KEGG pathway enrichment analyses of differential metabolites. **(B)** The top 20 are presented. The enrichment factor is the ratio of the number of DEGs to the number of total genes in a particular pathway. The dot size represents the number of differential metabolites, while colors correspond to the range of *p*-values.

**FIGURE 10:**
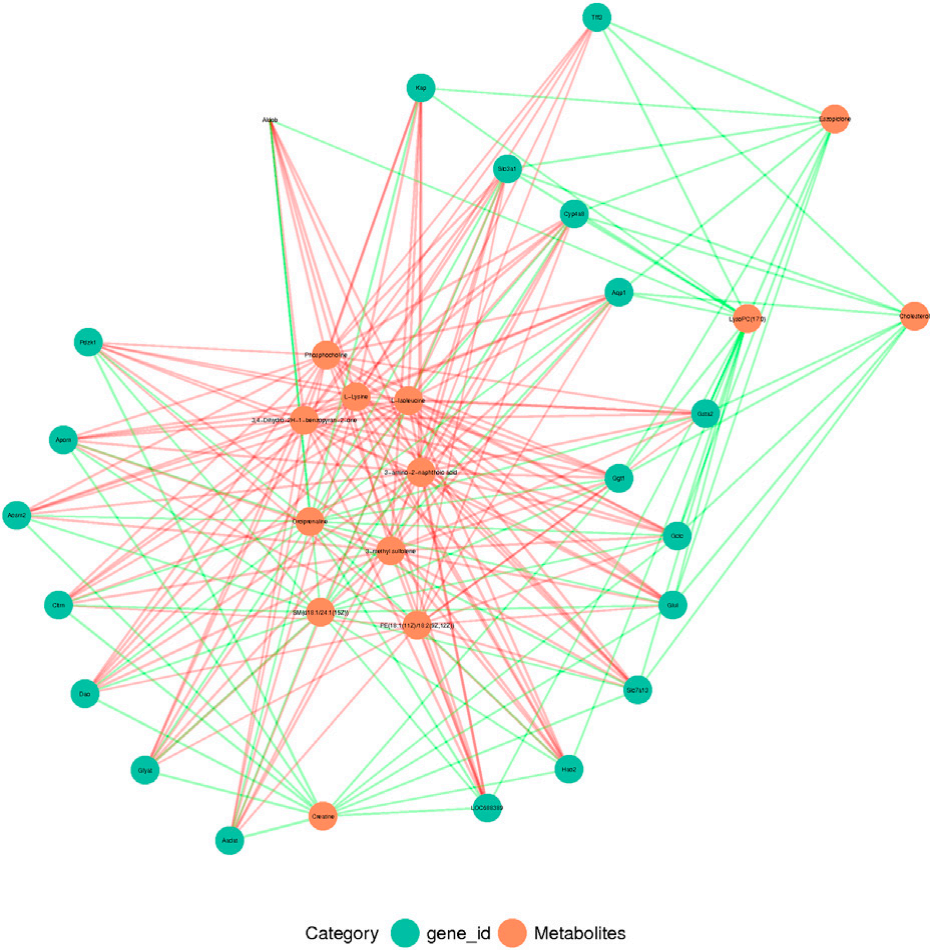
Conjoint analysis of transcriptome and metabolome in RIAKI. Green and red circles represent corresponding DEGs and metabolites, respectively. Green and red lines indicate positive and negative correlations between DEGs and metabolites, respectively.

The metabolomic analysis identified a total of 559 differential metabolites in the RM group compared with the CN group, and 262 differential metabolites with differential changes under NM intervention compared with RM. The KEGG enrichment analysis of differential metabolites showed the enriched differential metabolites were mainly involved in amino acid metabolism (lysine degradation, valine, leucine and isoleucine biosynthesis, arginine and proline metabolism, D-arginine and D-ornithine metabolism), choline metabolism in cancer, mTOR signaling pathway, glycerophospholipid metabolism, necrosis, apoptosis, synthesis and secretion of cortisol, vitamin digestion and absorption, and pyrimidine metabolism (Figure 9). Meanwhile, the integrated transcriptomics and metabolomics analyses revealed the association network of key DEGs and corresponding metabolites, among them*, Gclc* interacts with 16 differential metabolites, of which 8 metabolites were positively correlated with *Gclc*, while 8 metabolites were negatively correlated with *Gclc* (Table 3; Figure 10). This data suggests that the accumulation of *Gclc* may contribute to renal protection with NM in RIAKI.

**TABLE 3:** List of Gclc-related differential metabolites.

Metabolites	Correlation	*p*-value
PS(O-16:0/21:0)	0.768995	0.025725
3,4-Dihydro-2H-1-benzopyran-2-one	0.95275	0.000254
L-Isoleucine	0.937389	0.000585
3-amino-2-naphthoic acid	0.969783	6.74E-05
PE [18:1 (11Z)/18:2 (9Z,12Z)]	0.816883	0.01332
Phosphocholine	0.840225	0.009014
3-methyl sulfolene	0.931113	0.000776
L-Lysine	0.979215	2.21E-05
LysoPC(17:0)	−0.82278	0.012131
Cholesterol	−0.77034	0.025306
PC [18:0/18:2 (9Z, 12Z)]	−0.79634	0.018024
SM [d18:1/24:1 (15Z)]	−0.73429	0.038051
Creatine	−0.71213	0.047506
Eszopiclone	−0.78237	0.021746
9-tridecynoic acid	−0.72833	0.040467
Orciprenaline	−0.7953	0.018286

## Discussion

Our study demonstrated that NM exerts a protective effect against AKI in the context of RM. The further integrated transcriptomic and metabolomic analysis revealed that the DEGs regulated by NM were mainly implicated in the glutathione metabolism pathway, inflammatory response, and ferroptosis related pathways. More importantly, these pathways related genes *Anpep*, *Gclc*, *Ggt1*, *Mgst2*, *Cxcl1*, *Cxcl3*, *Rgn*, *Retn*, *Akr1c1*, and *Akr1c2* were highly regulated by NM. Based on our results, it can be speculated that the renal protective effect of NM on RIAKI is mainly produced through the regulation of glutathione metabolism, inflammatory response, and ferroptosis. Targeting these key pathways related genes may emerge as a potential molecular therapy for RIAKI.

Rhabdomyolysis is a common cause of AKI, and the mechanisms involved in RIAKI are complex. Therefore, further understanding of the potential molecular mechanism of RIAKI and the identification of new targets will facilitate the exploring of novel therapies for RIAKI. In the present study, we developed a glycerol induced experimental model of RIAKI in rats using well established methods (Al Asmari et al., 2017). Our model successfully replicated the pathogenesis of RIAKI characterized by the deterioration of kidney function, inflammation, cell apoptosis, and histopathological changes. We observed a significant increase in serum SCr and BUN over time after modeling, which indicated the progressive deterioration of kidney function. There was a significant increase in inflammatory cytokines and cell apoptosis in the modeling group. Our histological data also showed tubular pathological damages with a large amount of necrotic cells and myoglobin. Myoglobin is known to play an important role in the development of RIAKI (Gburek et al., 2003). Myoglobin is an iron and oxygen binding protein found generally in the muscle tissue of vertebrates and in almost all mammals. It has a higher binding affinity for oxygen than hemoglobin (David, 2000). Increased evidence has suggested that released myoglobin in circulation can activate the renin-angiotensin-aldosterone system, and trigger the release of vasopressin and nitric oxide deficiency, resulting in intrarenal vasoconstriction, tubular obstruction and direct tubule injury, and inflammation (Holt and Moore, 2001; Belliere et al., 2015). During RM, excessive amounts of myoglobin are released into circulation from muscle cells, and the free myoglobin is filtered by the glomerular filtration barrier and endocytosed by renal tubular cells *via* the epithelial endocytic receptors megalin and cubilin (Khan, 2009). Ferrous myoglobin inside tubular cells is oxidized to a ferric form, resulting in the production of a hydroxyl radical, followed by the transformation of ferric myoglobin into ferryl myoglobin through redox cycling, yielding of a radical species. These radical species lead to lipid peroxidation and excessive oxidative stress (Petejova and Martinek, 2014).

The clinical treatment for RIAKI is limited. The standard medical interventions are usually the management of underlying disease and initiation of conservative treatments, including massive hydration, urine alkalization and forced diuresis (Krouzecky et al., 2003). Currently, the core treatment is renal replacement therapy (RRT). It has been reported about 8%–65% of patients who develop RIAKI are in need of RRT (de Meijer et al., 2003). The possibility of myoglobin removal using either intermittent hemodialysis or CRRT in the case of RIAKI has been demonstrated in many studies (Amyot et al., 1999; Heyne et al., 2012). It has been shown in several retrospective studies that NM is safe and effective for patients with high bleeding risk in CRRT (Maruyama et al., 2011; Baek et al., 2012; Hwang et al., 2013), and does not affect patient mortality (Baek et al., 2012; Lee et al., 2014). Choi et al. Choi et al. (2015) reported that patients who received NM treatment had better survival rates at 30 and 90 days after CRRT initiation than when compared to the non-NM treatment group, however, the difference was not statistically significant. So far, there is very limited data regarding the protective effect of NM on RIAKI besides acting as an anticoagulant in CRRT. Our study found, for the first time, that NM intervention offers a beneficial effect in RIAKI in a rat model.

As is known there is excessive oxidation during RM induced renal injury (Moore et al., 1998; Giannoglou et al., 2007). Glutathione (GSH) metabolism is one of the most important antioxidant systems against oxidative stress, which plays an important role in regulating oxidative stress in many diseases, such as diabetes, stroke, and neurodegenerative disease etc. (Wu et al., 2004). The downregulation or deficiency of GSH has been associated with excessive oxidative stress in renal ischemic repercussion disease (Shang et al., 2016). Glutathione protects cellular components against lipid peroxidation, and DNA and protein damage through neutralizing hydrogen peroxide and reactive oxygen species (ROS) (Hanschmann et al., 2013) Cellular GSH is mostly present in the cytosol and can be oxidized by ROS into glutathione disulfide (GSSG). Therefore, the ratio of GSH/GSSG can indicate the cellular redox state. Glutamate-cysteine ligase (GCL) is the rate limiting enzyme for GSH synthesis. It consists of a catalytic subunit *Gclc* and a modifier subunit *Gclm* (Giordano et al., 2007). The catalytic *Gclc* subunit is essential for the enzymatic activity, while the modulatory *Gclm* subunit enhances *Gclc* catalytic efficiency. It has been reported that a lack of GCLM results in a reduction of GSH and consequent vulnerability to oxidative stress. The expression of GCL is induced by oxidative stress and this process can be regulated by some pivotal transcription factors, such as activator protein-1, nuclear factor-erythroid 2 related factor 2, and nuclear factor κB (NFκB) etc. (Lu, 2009). The amount of GCL subunits, the availability of substrates, and the extent of feedback inhibition of GCL by GSH control the overall rate of GSH synthesis. Botta et al. Botta et al. (2004) reported that overexpression of *Gclc* and GCLM in mouse liver hepatoma cells exhibits increased resistance to TNF-induced mitochondria injury and apoptosis. Tran’s study Tran et al. (2004) showed that adenoviral overexpression of the GSH catalytic subunit *Gclc* increases intracellular GSH levels and protects beta cells against oxidative stress. Therefore, the strategy favors the biosynthesis of GSH and can be an attractive antioxidant therapy. Boutaud et al. Boutaud et al. (2010) reported that acetaminophen attenuates RM induced renal failure by reducing radical species generated from redox cycling between ferric and ferryl myoglobin. Chander et al. Chander et al. (2003) showed that a natural antioxidant, catechin, can reduce the toxicity of circulating myoglobin in the kidney tissues, and protect the kidneys against myoglobinuria acute renal failure in rats. In line with these studies, our results found that NM protects the kidneys against RIAKI, which may be associated with the upregulation of *Gclc* through activating the glutathione metabolism pathway. This hypothesis was supported by our observation that NM treatment increased GSH/GSSG ratio significantly compared to RM group. In the meantime, we observed an increased expression of Ggt1 after NM intervention, which is consistent with a report that showed the protective action of GSH requires GSH hydrolysis catalyzed by Ggt1 (Paolicchi et al., 2003). Our conjoint analysis of the transcriptome and metabolome also showed that *Gclc* interacted with 16 key differential metabolites, which suggests *Gclc* may be the key gene implicated in the renal protective effect of NM in RIAKI. This provides a mechanistic basis for antioxidant therapy in RIAKI. The physiological function of Mgst2 as a mediator of oxidative stress and DNA damage in nonimmune cells has been well studied. Dvash et al. (2015)‘s study has shown that Mgst2 deficiency attenuates ER stress-triggered oxidative stress, DNA damage and apoptosis, and mouse morbidity in acute kidney injury. Consistent with this study, our data showed that NM treatment decreases the transcription level of Mgst2, which may contribute to renal protection.

It is known that inflammation plays a pivotal role in the pathogenesis of RIAKI and that the leukocytes, particularly macrophages, are involved in the development of RIAKI (Holt and Moore, 2001). Macrophage infiltration has been reported in RM patient’s kidney biopsy specimens and glycerol-induced AKI in rat models (Sinniah and Lye, 2000; Homsi et al., 2006). Myoglobin can stimulate proximal tubular cells to secrete macrophages and many molecules, such as high mobility group box proteins, uric acid, and microRNAs. These molecules are released from damaged muscle cells and activate the immune system, which lead to the activation of dendritic cells, Toll-like receptors, T-lymphocytes, macrophages, and NFκβ, and result in the increased generation of pro-inflammatory cytokines, such as IL-1 and TNF. Increased expression of adhesive molecules (ICAM-1, VCAM-1 etc.) also enhances the inflammatory response. Myoglobin-derived heme is another important molecular that influences the inflammatory reaction in the tubular endothelium and epithelium cells (Jang and Rabb, 2009; Panizo et al., 2015). Therefore, anti-inflammatory treatment can be a potential therapeutic strategy in RIAKI. Macrophage depletion has been reported to protect the kidneys against ischemia-reperfusion induced AKI (Ko et al., 2008). A phosphodiesterase inhibitor, pentoxifylline, was shown to decrease inflammation through the inhibition of TNF-α and leukotriene production in RIAKI (Plotnikov et al., 2009). In agreement with the above studies, our results showed that NM offers kidney protection through reducing the pro-inflammatory cytokines IL-6 and TNF-α in RIAKI. Our data showed that NM also upregulated the anti-inflammatory gene *Rgn*, which is consistent with the report that *Rgn* may act as a suppressor of inflammation in macrophage-infiltrated adipocyte tissue (Murata et al., 2020). Surprisingly, we found that NM induced the expression of the pro-inflammatory chemokine Cxcl13. However, the underlying cause is still not clear. Nevertheless, the effect of NM induced upregulation of Cxcl13 was overwhelmed by the NM mediated renoprotection. In the setting of RM, lipid peroxidation and oxidative stress can stimulate the release of cytochrome c from the mitochondria which lead to the activation of caspase 1 and 3, resulting in apoptosis (Panizo et al., 2015). However, other types of cell death may also be involved in RIAKI. It has been reported that there was no change in creatinine levels in mice that received specific apoptosis inhibitors (Homsi et al., 2006). Guerrero-Hue et al. (2019) demonstrated another type of nonapoptotic cell death. Ferroptosis plays a pivotal role in kidney injury associated with RM in *vitro* myoglobin stimulated renal tubular cells and in the mouse model of RIAKI, and Melania et al. found that ferroptosis is consistent with the increased lipid peroxidation and iron accumulation in the context of RM. More importantly, they found that the inhibition of ferroptosis attenuated kidney dysfunction, whereas no effects were observed with the inhibition of apoptosis or necroptosis, which implicates a prominent contribution of ferroptosis rather than apoptosis to the RIAKI. Many other studies have also reported that ferroptosis contributes to kidney injury caused by cisplatin, folic acid, and ischemia-reperfusion injury in experimental models (Linkermann et al., 2014a; Linkermann et al., 2014b; Martin-Sanchez et al., 2017). Our study investigated the ferroptosis related protein GPX4 in the context of RIAKI. As is known, GPX4 is a key GSH-utilizing enzyme which counteracts lipoxygenase activity and phospholipid/cardiolipin oxidation (Conrad and Friedmann Angeli, 2015). Inhibition of GPX4 causes the accumulation of iron-dependent lipid peroxidation, which ultimately leads to ferroptotic cell death (Chen et al., 2020). Friedmann Angeli et al. (2014) reported that GPX4 knockout in tubular cells triggers ferroptosis and substantial cell death in acute kidney injury. In line with these studies, our data showed the repression of GPX4 and increased accumulation of iron in RIAKI, while the expression of GPX4 increased significantly after NM treatment accompanied with decreased iron levels, which supports the idea that ferroptosis may also be involved in RIAKI and can be regulated by NM. Meanwhile, we observed that NM intervention mitigates apoptosis induced by RIAKI. Considering the TUNEL staining used in our study is not specific for apoptosis, we also investigated the effect of NM on MMP, and found NM treatment attenuates the loss in MMP in response to RIAKI.

The aldo-keto reductases (AKRs) gene family has been reported to inhibit ferroptosis through potentiating the metabolization of lipid peroxides by catalyzing aldehydes and ketones to alcohols (Dixon et al., 2012). Gagliardi et al. (2019) study showed that AKR1C1/3 degrades the 12/15-LOX-generated lipid peroxides, thus resulting in ferroptotic cell death resistance. It has been reported that AKR1C1 protects cancer cells from ferroptosis and is considered to be the ferroptosis-protective gene (Wohlhieter et al., 2020). These studies support the notion that AKR1C1 plays a role in ferroptosis. Our data also showed that AKR Family one Member C1 (Akr1c1) and Akr1c2 were both highly regulated by NM, which suggests that the inhibition of ferroptosis contributes to the NM mediated renal protection in RIAKI.

We appreciate the limitations of our study. First, we treated the RIAKI rats with NM at a dose of 1 mg/kg and did not examine the dose-response relationship of the effect of NM on RIAKI. Second, we administered NM prior to the induction of RIAKI, which is not amenable in clinical practice. Thus, the therapeutic effect of NM on RIAKI remains to be elucidated in further studies. Another limitation is that the sample size of our study was relatively small, which may cause a certain degree of sampling bias. Finally, our study did not define the molecular functions of the key DEGs and metabolites identified and their possible mechanisms by which RIAKI is attenuated with treatment of NM. Rather, our work provides the transcriptomic and metabolomic profiling and framework from which the key molecules can be prioritized for future mechanistic studies.

## Conclusion

Our study comprehensively analyzed the changes in the abundance of genes and metabolites regulated by NM during RIAKI. The results revealed that NM broadly activates glutathione metabolism, inflammatory, and ferroptosis associated signaling pathway, which leads to accumulation of antioxidant, anti-inflammatory, and anti-ferroptosis related metabolites and genes. Our findings provided insight into the regulatory mechanism of NM mediated renal protection, which facilitates the exploration and translation of therapeutic or diagnostic properties of these candidate genes to the clinic. However, the functional characterization of the identified DEGs in this study still needs to be further investigated.

## Data Availability

The datasets presented in this study can be found in online repositories. The names of the repository/repositories and accession number(s) can be found below: https://www.ncbi.nlm.nih.gov/bioproject/PRJNA851578.

## Footnotes

1 Shifu C., Yanqing Z., Yaru C., et al. fastp: An ultra-fast all-in-one FASTQ preprocessor[J]. Bioinformatics, 2018, 34 (17): i884–i890.

2 Kim D., Langmead B. zz and Salzberg S. L. HISAT: A fast spliced aligner with low memory requirements. *Nature Methods* 2015; 12 (4): 357–360.

3 Roberts A., Trapnell C., Donaghey J., et al. Improving RNA-Seq expression estimates by correcting for fragment bias. Genome Biology 2011; 12 (3): R22.

4 Anders S, Pyl PT and Huber W. HTSeq—a Python framework to work with high-throughput sequencing data. Bioinformatics 2015; 31 (2): 166–169.

5 Love M. I., Huber W., Anders S. Moderated estimation of fold change and dispersion for RNA-seq data with DESeq2[J]. Genome Biology, 2014.

6 The Gene Ontology Resource: 20 years and still going strong[J]. Nucleic Acids Res. January 2019; 47 (D1): D330–D338.

7 Kanehisa M., Araki M., Goto S., et al. KEGG for linking genomes to life and the environment[J]. Nucleic acids research, 2008, 36 (suppl 1): D480–D484.

## References

[B1] Al Asmari A. K. Al Sadoon K. T. Obaid A. A. Yesunayagam D. Tariq M. (2017). Protective effect of quinacrine against glycerol-induced acute kidney injury in rats. BMC Nephrol. 18 (1), 41. 10.1186/s12882-017-0450-8 28129740 PMC5273840

[B2] Amyot S. L. Leblanc M. Thibeault Y. Geadah D. Cardinal J. (1999). Myoglobin clearance and removal during continuous venovenous hemofiltration. Intensive Care Med. 25 (10), 1169–1172. 10.1007/s001340051031 10551978

[B3] Baek N. N. Jang H. R. Huh W. Kim Y. G. Kim D. J. Oh H. Y. (2012). The role of nafamostat mesylate in continuous renal replacement therapy among patients at high risk of bleeding. Ren. Fail. 34 (3), 279–85. 10.3109/0886022x.2011.647293 22251267

[B4] Bagley W. H. Yang H. Shah K. H. (2007). Rhabdomyolysis. Intern. Emerg. Med. 2 (3), 210–218. 10.1007/s11739-007-0060-8 17909702

[B5] Belliere J. Casemayou A. Ducasse L. Zakaroff-Girard A. Martins F. Iacovoni J. S. (2015). Specific macrophage subtypes influence the progression of rhabdomyolysis-induced kidney injury. J. Am. Soc. Nephrol. 26 (6), 1363–1377. 10.1681/asn.2014040320 25270069 PMC4446873

[B6] Bosch X. Poch E. Grau J. M. (2009). Rhabdomyolysis and acute kidney injury. N. Engl. J. Med. 361 (1), 62–72. 10.1056/NEJMra0801327 19571284

[B7] Botta D. Franklin C. C. White C. C. Krejsa C. M. Dabrowski M. J. Pierce R. H. (2004). Glutamate-cysteine ligase attenuates TNF-induced mitochondrial injury and apoptosis. Free Radic. Biol. Med. 37 (5), 632–642. 10.1016/j.freeradbiomed.2004.05.027 15288121

[B8] Boutaud O. Moore K. P. Reeder B. J. Harry D. Howie A. J. Wang S. (2010). Acetaminophen inhibits hemoprotein-catalyzed lipid peroxidation and attenuates rhabdomyolysis-induced renal failure. Proc. Natl. Acad. Sci. U. S. A. 107 (6), 2699–2704. 10.1073/pnas.0910174107 20133658 PMC2823910

[B9] Cervellin G. Comelli I. Lippi G. (2010). Rhabdomyolysis: Historical background, clinical, diagnostic and therapeutic features. Clin. Chem. Lab. Med. 48 (6), 749–756. 10.1515/cclm.2010.151 20298139

[B10] Chander V. Singh D. Chopra K. (2003). Catechin, a natural antioxidant protects against rhabdomyolysis-induced myoglobinuric acute renal failure. Pharmacol. Res. 48 (5), 503–509. 10.1016/s1043-6618(03)00207-x 12967597

[B11] Chatzizisis Y. S. Misirli G. Hatzitolios A. I. Giannoglou G. D. (2008). The syndrome of rhabdomyolysis: Complications and treatment. Eur. J. Intern. Med. 19 (8), 568–574. 10.1016/j.ejim.2007.06.037 19046720

[B12] Chen X. Yu C. Kang R. Tang D. (2020). Iron metabolism in ferroptosis. Front. Cell Dev. Biol. 8, 590226. 10.3389/fcell.2020.590226 33117818 PMC7575751

[B13] Choi J. Y. Kang Y. J. Jang H. M. Jung H. Y. Cho J. H. Park S. H. (2015). Nafamostat mesilate as an anticoagulant during continuous renal replacement therapy in patients with high bleeding risk: A randomized clinical trial. Med. Baltim. 94 (52), e2392. 10.1097/md.0000000000002392 PMC5291631 26717390

[B15] Conrad M. Friedmann Angeli J. P. (2015). Glutathione peroxidase 4 (Gpx4) and ferroptosis: what's so special about it? Mol. Cell. Oncol. 2 (3), e995047. 10.4161/23723556.2014.995047 27308484 PMC4905320

[B17] David W. S. (2000). Myoglobinuria. Neurol. Clin. 18 (1), 215–243. 10.1016/s0733-8619(05)70187-0 10658177

[B18] de Meijer A. R. Fikkers B. G. de Keijzer M. H. van Engelen B. G. Drenth J. P. (2003). Serum creatine kinase as predictor of clinical course in rhabdomyolysis: A 5-year intensive care survey. Intensive Care Med. 29 (7), 1121–1125. 10.1007/s00134-003-1800-5 12768237

[B19] Dixon S. J. Lemberg K. M. Lamprecht M. R. Skouta R. Zaitsev E. M. Gleason C. E. (2012). Ferroptosis: An iron-dependent form of nonapoptotic cell death. Cell 149 (5), 1060–1072. 10.1016/j.cell.2012.03.042 22632970 PMC3367386

[B20] Dvash E. Har-Tal M. Barak S. Meir O. Rubinstein M. (2015). Leukotriene C4 is the major trigger of stress-induced oxidative DNA damage. Nat. Commun. 6, 10112. 10.1038/ncomms10112 26656251 PMC4682057

[B21] Friedmann Angeli J. P. Schneider M. Proneth B. Tyurina Y. Y. Tyurin V. A. Hammond V. J. (2014). Inactivation of the ferroptosis regulator Gpx4 triggers acute renal failure in mice. Nat. Cell Biol. 16 (12), 1180–1191. 10.1038/ncb3064 25402683 PMC4894846

[B22] Gagliardi M. Cotella D. Santoro C. Corà D. Barlev N. A. Piacentini M. (2019). Aldo-keto reductases protect metastatic melanoma from ER stress-independent ferroptosis. Cell Death Dis. 10 (12), 902. 10.1038/s41419-019-2143-7 31780644 PMC6883066

[B23] Gburek J. Birn H. Verroust P. J. Goj B. Jacobsen C. Moestrup S. K. (2003). Renal uptake of myoglobin is mediated by the endocytic receptors megalin and cubilin. Am. J. Physiol. Ren. Physiol. 285 (3), F451–F458. 10.1152/ajprenal.00062.2003 12724130

[B24] Giannoglou G. D. Chatzizisis Y. S. Misirli G. (2007). The syndrome of rhabdomyolysis: Pathophysiology and diagnosis. Eur. J. Intern. Med. 18 (2), 90–100. 10.1016/j.ejim.2006.09.020 17338959

[B25] Giordano G. Afsharinejad Z. Guizzetti M. Vitalone A. Kavanagh T. J. Costa L. G. (2007). Organophosphorus insecticides chlorpyrifos and diazinon and oxidative stress in neuronal cells in a genetic model of glutathione deficiency. Toxicol. Appl. Pharmacol. 219 (2-3), 181–189. 10.1016/j.taap.2006.09.016 17084875

[B26] Guerrero-Hue M. García-Caballero C. Palomino-Antolín A. Rubio-Navarro A. Vázquez-Carballo C. Herencia C. (2019). Curcumin reduces renal damage associated with rhabdomyolysis by decreasing ferroptosis-mediated cell death. Faseb J. 33 (8), 8961–8975. 10.1096/fj.201900077R 31034781

[B27] Hanschmann E. M. Godoy J. R. Berndt C. Hudemann C. Lillig C. H. (2013). Thioredoxins, glutaredoxins, and peroxiredoxins--molecular mechanisms and health significance: From cofactors to antioxidants to redox signaling. Antioxid. Redox Signal. 19 (13), 1539–1605. 10.1089/ars.2012.4599 23397885 PMC3797455

[B28] Heyne N. Guthoff M. Krieger J. Haap M. Häring H. U. (2012). High cut-off renal replacement therapy for removal of myoglobin in severe rhabdomyolysis and acute kidney injury: A case series. Nephron. Clin. Pract. 121 (3-4), c159–c164. 10.1159/000343564 23327834

[B29] Holt S. G. Moore K. P. (2001). Pathogenesis and treatment of renal dysfunction in rhabdomyolysis. Intensive Care Med. 27 (5), 803–811. 10.1007/s001340100878 11430535

[B30] Homsi E. Janino P. de Faria J. B. (2006). Role of caspases on cell death, inflammation, and cell cycle in glycerol-induced acute renal failure. Kidney Int. 69 (8), 1385–1392. 10.1038/sj.ki.5000315 16557226

[B31] Hwang S. D. Hyun Y. K. Moon S. J. Lee S. C. Yoon S. Y. (2013). Nafamostat mesilate for anticoagulation in continuous renal replacement therapy. Int. J. Artif. Organs 36 (3), 208–216. 10.5301/ijao.5000191 23404639

[B32] Jang H. R. Rabb H. (2009). The innate immune response in ischemic acute kidney injury. Clin. Immunol. 130 (1), 41–50. 10.1016/j.clim.2008.08.016 18922742 PMC2646108

[B33] Khan F. Y. (2009). Rhabdomyolysis: A review of the literature. Neth. J. Med. 67 (9), 272–283. 19841484

[B34] Ko G. J. Boo C. S. Jo S. K. Cho W. Y. Kim H. K. (2008). Macrophages contribute to the development of renal fibrosis following ischaemia/reperfusion-induced acute kidney injury. Nephrol. Dial. Transpl. 23 (3), 842–852. 10.1093/ndt/gfm694 17984109

[B35] Krouzecký A. Matĕjovic M. Rokyta R. Jr. Novák I. Krouzecky A. (2003). Rhabdomyolysis--development, causes, sequelae and therapy. Vnitr. Lek. 49 (8), 668–672. 14518093

[B36] Lee Y. K. Lee H. W. Choi K. H. Kim B. S. (2014). Ability of nafamostat mesilate to prolong filter patency during continuous renal replacement therapy in patients at high risk of bleeding: A randomized controlled study. PLoS One 9 (10), e108737. 10.1371/journal.pone.0108737 25302581 PMC4193755

[B37] Leuthold P. Schwab M. Hofmann U. Winter S. Rausch S. Pollak M. N. (2018). Simultaneous extraction of RNA and metabolites from single kidney tissue specimens for combined transcriptomic and metabolomic profiling. J. Proteome Res. 17 (9), 3039–3049. 10.1021/acs.jproteome.8b00199 30091608

[B38] Linkermann A. Chen G. Dong G. Kunzendorf U. Krautwald S. Dong Z. (2014a). Regulated cell death in AKI. J. Am. Soc. Nephrol. 25 (12), 2689–2701. 10.1681/asn.2014030262 24925726 PMC4243360

[B39] Linkermann A. Skouta R. Himmerkus N. Mulay S. R. Dewitz C. De Zen F. (2014b). Synchronized renal tubular cell death involves ferroptosis. Proc. Natl. Acad. Sci. U. S. A. 111 (47), 16836–16841. 10.1073/pnas.1415518111 25385600 PMC4250130

[B40] Lu S. C. (2009). Regulation of glutathione synthesis. Mol. Asp. Med. 30 (1-2), 42–59. 10.1016/j.mam.2008.05.005 PMC2704241 18601945

[B41] Martin-Sanchez D. Ruiz-Andres O. Poveda J. Carrasco S. Cannata-Ortiz P. Sanchez-Niño M. D. (2017). Ferroptosis, but not necroptosis, is important in nephrotoxic folic acid-induced AKI. J. Am. Soc. Nephrol. 28 (1), 218–229. 10.1681/asn.2015121376 27352622 PMC5198282

[B42] Maruyama H. Miyakawa Y. Gejyo F. Arakawa M. (1996). Anaphylactoid reaction induced by nafamostat mesilate in a hemodialysis patient. Nephron 74 (2), 468–469. 10.1159/000189371 8893192

[B43] Maruyama Y. Yoshida H. Uchino S. Yokoyama K. Yamamoto H. Takinami M. (2011). Nafamostat mesilate as an anticoagulant during continuous veno-venous hemodialysis: A three-year retrospective cohort study. Int. J. Artif. Organs 34 (7), 571–576. 10.5301/ijao.2011.8535 21786254

[B44] McMahon G. M. Zeng X. Waikar S. S. (2013). A risk prediction score for kidney failure or mortality in rhabdomyolysis. JAMA Intern. Med. 173 (19), 1821–1828. 10.1001/jamainternmed.2013.9774 24000014 PMC5152583

[B45] Moore K. P. Holt S. G. Patel R. P. Svistunenko D. A. Zackert W. Goodier D. (1998). A causative role for redox cycling of myoglobin and its inhibition by alkalinization in the pathogenesis and treatment of rhabdomyolysis-induced renal failure. J. Biol. Chem. 273 (48), 31731–31737. 10.1074/jbc.273.48.31731 9822635

[B46] Murata T. Yamaguchi M. Kohno S. Takahashi C. Risa W. Hatori K. (2020). Regucalcin enhances adipocyte differentiation and attenuates inflammation in 3T3-L1 cells. FEBS Open Bio 10 (10), 1967–1984. 10.1002/2211-5463.12947 PMC7530391 32783343

[B47] Na K. R. Choi H. Jeong J. Y. Lee K. W. Chang Y. K. Choi D. E. (2016). Nafamostat mesilate attenuates ischemia-reperfusion-induced renal injury. Transpl. Proc. 48 (6), 2192–2199. 10.1016/j.transproceed.2016.03.050 27569970

[B48] Nakae H. Tajimi K. (2003). Pharmacokinetics of nafamostat mesilate during continuous hemodiafiltration with a polyacrylonitrile membrane. Ther. Apher. Dial. 7 (5), 483–485. 10.1046/j.1526-0968.2003.00088.x 14708904

[B49] Ohtake Y. Hirasawa H. Sugai T. Oda S. Shiga H. Matsuda K. (1991). Nafamostat mesylate as anticoagulant in continuous hemofiltration and continuous hemodiafiltration. Contrib. Nephrol. 93, 215–217. 10.1159/000420222 1666354

[B50] Panizo N. Rubio-Navarro A. Amaro-Villalobos J. M. Egido J. Moreno J. A. (2015). Molecular mechanisms and novel therapeutic approaches to rhabdomyolysis-induced acute kidney injury. Kidney Blood Press. Res. 40 (5), 520–532. 10.1159/000368528 26512883

[B51] Paolicchi A. Sotiropuolou M. Perego P. Daubeuf S. Visvikis A. Lorenzini E. (2003). gamma-Glutamyl transpeptidase catalyses the extracellular detoxification of cisplatin in a human cell line derived from the proximal convoluted tubule of the kidney. Eur. J. Cancer 39 (7), 996–1003. 10.1016/s0959-8049(03)00067-4 12706370

[B52] Petejova N. Martinek A. (2014). Acute kidney injury due to rhabdomyolysis and renal replacement therapy: A critical review. Crit. Care 18 (3), 224. 10.1186/cc13897 25043142 PMC4056317

[B53] Plotnikov E. Y. Chupyrkina A. A. Pevzner I. B. Isaev N. K. Zorov D. B. (2009). Myoglobin causes oxidative stress, increase of NO production and dysfunction of kidney's mitochondria. Biochim. Biophys. Acta 1792 (8), 796–803. 10.1016/j.bbadis.2009.06.005 19545623

[B54] Shang Y. Siow Y. L. Isaak C. K. O K. (2016). Downregulation of glutathione biosynthesis contributes to oxidative stress and liver dysfunction in acute kidney injury. Oxid. Med. Cell. Longev. 2016, 1–13. 10.1155/2016/9707292 PMC5107229 27872680

[B55] Shapiro M. L. Baldea A. Luchette F. A. (2012). Rhabdomyolysis in the intensive care unit. J. Intensive Care Med. 27 (6), 335–342. 10.1177/0885066611402150 21436168

[B56] Simpson J. P. Taylor A. Sudhan N. Menon D. K. Lavinio A. (2016). Rhabdomyolysis and acute kidney injury: Creatine kinase as a prognostic marker and validation of the McMahon score in a 10-year cohort: A retrospective observational evaluation. Eur. J. Anaesthesiol. 33 (12), 906–912. 10.1097/eja.0000000000000490 27259093

[B57] Sinniah R. Lye W. (2000). Acute renal failure from myoglobinuria secondary to myositis from severe falciparum malaria. Am. J. Nephrol. 20 (4), 339–343. 10.1159/000013611 10970990

[B58] Tran P. O. Parker S. M. LeRoy E. Franklin C. C. Kavanagh T. J. Zhang T. (2004). Adenoviral overexpression of the glutamylcysteine ligase catalytic subunit protects pancreatic islets against oxidative stress. J. Biol. Chem. 279 (52), 53988–53993. 10.1074/jbc.M404809200 15485876

[B59] van de Wetering J. Westendorp R. G. van der Hoeven J. G. Stolk B. Feuth J. D. Chang P. C. (1996). Heparin use in continuous renal replacement procedures: The struggle between filter coagulation and patient hemorrhage. J. Am. Soc. Nephrol. 7 (1), 145–150. 10.1681/asn.v71145 8808122

[B60] Vanholder R. Van Biesen W. Hoste E. Lameire N. (2011). Pro/con debate: Continuous versus intermittent dialysis for acute kidney injury: A never-ending story yet approaching the finish? Crit. Care 15 (1), 204. 10.1186/cc9345 21345275 PMC3222013

[B61] Webb A. R. Mythen M. G. Jacobson D. Mackie I. J. (1995). Maintaining blood flow in the extracorporeal circuit: Haemostasis and anticoagulation. Intensive Care Med. 21 (1), 84–93. 10.1007/bf02425162 7560483

[B62] Wohlhieter C. A. Richards A. L. Uddin F. Hulton C. H. Quintanal-Villalonga À. Martin A. (2020). Concurrent mutations in STK11 and KEAP1 promote ferroptosis protection and SCD1 dependence in lung cancer. Cell Rep. 33 (9), 108444. 10.1016/j.celrep.2020.108444 33264619 PMC7722473

[B63] Wu G. Fang Y. Z. Yang S. Lupton J. R. Turner N. D. (2004). Glutathione metabolism and its implications for health. J. Nutr. 134 (3), 489–492. 10.1093/jn/134.3.489 14988435

[B64] Zimmerman J. L. Shen M. C. (2013). Rhabdomyolysis. Chest 144 (3), 1058–1065. 10.1378/chest.12-2016 24008958

